# Developmental Exposure of Rats to Chlorpyrifos Leads to Behavioral Alterations in Adulthood, Involving Serotonergic Mechanisms and Resembling Animal Models of Depression

**DOI:** 10.1289/ehp.7867

**Published:** 2005-02-02

**Authors:** Justin E. Aldridge, Edward D. Levin, Frederic J. Seidler, Theodore A. Slotkin

**Affiliations:** ^1^Department of Pharmacology and Cancer Biology, and; ^2^Department of Psychiatry and Behavioral Sciences, Duke University Medical Center, Durham, North Carolina, USA

**Keywords:** animal models, behavioral deficits, brain development, chlorpyrifos, depression, organophosphates, serotonin

## Abstract

Developmental exposure to chlorpyrifos (CPF) causes persistent changes in serotonergic (5HT) systems. We administered 1 mg/kg/day CPF to rats on postnatal days 1–4, a regimen below the threshold for systemic toxicity. When tested in adulthood, CPF-exposed animals showed abnormalities in behavioral tests that involve 5HT mechanisms. In the elevated plus maze, males treated with CPF spent more time in the open arms, an effect seen with 5HT deficiencies in animal models of depression. Similarly, in an anhedonia test, the CPF-exposed group showed a decreased preference for chocolate milk versus water. Developmental CPF exposure also has lasting effects on cognitive function. We replicated our earlier finding that developmental CPF exposure ablates the normal sex differences in 16-arm radial maze learning and memory: during acquisition training, control male rats typically perform more accurately than do control females, but CPF treatment eliminated this normal sex difference. Females exposed to CPF showed a reduction in working and reference memory errors down to the rate of control males. Conversely, CPF-exposed males exhibited an increase in working and reference memory errors. After radial-arm acquisition training, we assessed the role of 5HT by challenging the animals with the 5HT_2_ receptor antagonist ketanserin. Ketanserin did not affect performance in controls but elicited dose-dependent increases in working and reference memory errors in the CPF group, indicating an abnormal dependence on 5HT systems. Our results indicate that neonatal CPF exposures, classically thought to be subtoxic, produce lasting changes in 5HT-related behaviors that resemble animal models of depression.

Chlorpyrifos (CPF), one of the most widely used organophosphate pesticides, has undergone increased restriction of use in the United States because of developmental neurotoxicity [U.S. Environmental Protection Agency (EPA) 2000, 2002]. Many animal studies have focused on long-term cholinergic deficits and related behaviors, given the acknowledged role of cholinesterase inhibition and cholinergic hyperstimulation in the systemic toxicity of organophosphates (Barone et al. 2000; Clegg and van Gemert 1999a, 1999b; Mileson et al. 1998; Pope 1999; Slotkin 1999, 2004). However, in the developing brain, CPF affects many of the basic processes of neural cell development through mechanisms that are not reflective of cholinesterase inhibition or cholinergic mechanisms, eliciting widespread disruption of neural cell replication and differentiation, axonogenesis and synaptogenesis, and synaptic function (Barone et al. 2000; Casida and Quistad 2004; Gupta 2004; Pope 1999; Slotkin 1999, 2004). Importantly, most of these effects are seen at CPF exposures below the threshold for systemic toxicity and even below the threshold for cholinesterase inhibition (Barone et al. 2000; Casida and Quistad 2004; Gupta 2004; Pope 1999; Qiao et al. 2002, 2003; Schuh et al. 2002; Slotkin 1999, 2004) and are major contributors to persistent neurobehavioral defects (Icenogle et al. 2004; Levin et al. 2001, 2002).

In fact, long-term alterations after fetal or neonatal CPF exposure are not confined to cholinergic systems but rather involve a wide variety of neurotransmitters and, notably, serotonin (5HT) (Aldridge et al. 2003, 2004; Dam et al. 1999; Raines et al. 2001; Slotkin 1999, 2004; Slotkin et al. 2002). Deficiencies in 5HT systems are a hallmark of human depression, and the most effective therapies use drugs designed to restore 5HT synaptic function (Maes and Meltzer 1995). In the present study, we evaluated the effects of neonatal CPF exposure on behaviors in adulthood that are well established in animal models of depression to reflect 5HT synaptic dysfunction (Cairncross 1984; Cryan et al. 1999; Harkin et al. 1999; Jesberger and Richardson 1985; Kelly et al. 1997; Leonard and Tuite 1981; Richardson 1991; Slotkin et al. 1999). In the elevated plus maze, a decrease in 5HT input suppresses the normal adaptive fear response (Kusserow et al. 2004), thereby increasing the amount of time spent in the open arms, an effect reversed by administration of 5HT agonists or 5HT reuptake inhibitors (Kelly et al. 1997; Mar et al. 2000; Martin et al. 1998; McGrath and Norman 1999). Similarly, anhedonia can be tested by the loss of preference for chocolate milk compared with water (Cairncross 1984; Jesberger and Richardson 1985; Kelly et al. 1997; Leonard and Tuite 1981; Pucilowski et al. 1993; Richardson 1991; Slotkin et al. 1999), a response that again is typical of a deficit in 5HT function (Juckel et al. 2003). Finally, because neonatal CPF exposure has persistent effects on cognitive function (Icenogle et al. 2004; Levin et al. 2001, 2002), we evaluated learning and memory in the 16-arm radial maze (Icenogle et al. 2004; Levin et al. 2001, 2002), assessing both basal performance and the effects of a pharmacologic challenge with a 5HT_2_ antagonist, ketanserin, to establish a mechanistic link between effects on 5HT and resultant cognitive alterations. Studies were conducted after CPF exposure of neonatal rats on postnatal days (PND)1–4, a period of heightened sensitivity to long-term effects on biomarkers of 5HT synaptic function (Aldridge et al. 2003, 2004).

## Materials and Methods

### Animal treatments.

All experiments were carried out in accordance with the *Guide for the Care and Use of Laboratory Animals* (Institute of Laboratory Animal Resources 1996) as adopted and promulgated by the National Institutes of Health. Timed-pregnant Sprague-Dawley rats (Charles River, Raleigh, NC) were housed in breeding cages, with a 12-hr light/dark cycle and free access to food and water. On the day of birth, all pups were randomized and redistributed to the dams with a litter size of 10 to maintain a standard nutritional status. Randomization within the respective treatment groups was repeated at intervals of several days; in addition, dams were rotated among litters to distribute any maternal caretaking differences randomly across litters and treatment groups. CPF (Chem Service, West Chester, PA) was dissolved in dimethylsulfoxide to provide consistent absorption (Whitney et al. 1995) and was injected subcutaneously at a dose of 1 mg/kg in a volume of 1 mL/kg once daily on PND1–4; control animals received equivalent injections of the dimethylsulfoxide vehicle. This regimen has been shown previously to produce neurotoxicity in developing rat brain, including lasting alterations in biomarkers of 5HT synaptic function, without eliciting growth retardation or any other signs of systemic toxicity (Aldridge et al. 2003, 2004). Indeed, neonatal brain cholinesterase inhibition is only about 25% (Song et al. 1997), well below the 70% threshold necessary for symptoms of cholinergic hyperstimulation (Clegg and van Gemert 1999a), thus resembling the nonsymptomatic exposures reported in pregnant women (De Peyster et al. 1993). Moreover, the dose used here is well within the range of typical fetal and childhood exposures after routine application (Gurunathan et al. 1998; Ostrea et al. 2002). Animals were weaned on PND21.

All behavioral testing was carried out during the dark phase, the most active period, but in lighted environments so that the rats could access the visual cues necessary to perform the tasks. Tests were performed on nine rats per sex per treatment group (36 animals in total), using no more than one male and one female from each litter. Beginning on PND47, the light/dark cycle was shifted by 6 hr (lights on at 0000 hr), and assessments were begun with the elevated plus maze (PND52–53) and the chocolate milk consumption test (PND54). On PND57, the light/dark cycle was shifted an additional 6 hr (lights on at 1800 hr), and radial-arm maze training was conducted during a 5-week period beginning on PND64. Ketanserin challenges in the radial-arm maze were undertaken in weeks 16–17. This sequence was designed to permit multiple behavioral tests to be conducted without significant carryover of effects from one test to the next (Icenogle et al. 2004; Levin et al. 2001, 2002; Slotkin et al. 1999). Tests were videotaped and scored by a trained observer who was blinded to the animal treatments.

### Elevated plus maze.

The maze was constructed of black-painted wood with arms 55 cm long and 10.2 cm wide, placed 50.8 cm above the floor. Two opposed arms had walls that were 15.2 cm tall, whereas the other two arms, set at 90° to walled arms, had smaller railings, only 2 cm tall. Rats were placed in the center area and allowed to roam freely for a total of 300 sec, and the time spent in the different types of arms was recorded, along with the number of times the animals crossed the center.

### Chocolate milk preference.

One hour after the start of the dark cycle, subjects were placed in a novel cage without food available and were presented with a choice of bottles containing water or chocolate milk. Consumption of each was measured after a single, 2-hr test session (Pucilowski et al. 1993; Slotkin et al. 1999).

### Radial-arm maze training.

During the period in which animals were trained in the radial-arm maze, they had free access to water, but food was restricted to approximately 15 g/day to provide an adequate stimulus to seek the food reward they obtained in the maze. The maze, made from black-painted wood, had a central platform 50 cm in diameter, elevated 30 cm from the floor, containing sixteen 10 × 60 cm arms projecting radially, each with a food cup 2 cm from the distal end. Tests were conducted in a quiet room with multiple extramaze visual cues that always remained in the same locations relative to the maze. The rats were familiarized with the food reinforcement by placing each rat in an opaque cylinder in the middle of the maze with a sugar-coated cereal (Froot Loops halves; Kellogg Co., Battle Creek, MI) and giving them up to 5 min to eat all of the pieces. Radial-arm maze training was then carried out 3 days/week for a total of 18 sessions per rat, grouped into blocks of three consecutive sessions for analysis. For each rat, the same 12 arms were baited at the beginning of each session, leaving the same four arms always unbaited; the array of baited and unbaited arms differed among rats. To begin each session, the rat was placed in an opaque cylinder in the middle of the maze for 10 sec to allow for orientation and to avoid bias as to which arm would be entered first. Timing began when the cylinder was removed, and the rat was allowed to roam freely about the maze for 10 min or until all 12 baited arms had been entered. Arm choice was recorded if all four paws crossed the threshold of the arm, and a note was made whether the reinforcement was eaten. The arms were not rebaited, so repeated entries into an arm were not rewarded and were counted as errors of working memory (Icenogle et al. 2004; Levin et al. 2001, 2002). Entries into arms that were never baited were recorded as reference memory errors. The response latency (time per entry) was calculated by dividing the total time of the session by the number of arms entered.

### Ketanserin challenges in the radial-arm maze.

After the 18 sessions of radial-arm maze training, the rats were tested by challenge with ketanserin, a 5HT_2_ receptor antagonist, to determine their reliance on 5HT mechanisms for memory. Twenty minutes before the test session, ketanserin was administered at subcutaneous doses of 0, 0.5, 1, or 2 mg/kg in 1 mL/kg isotonic saline vehicle. The different doses were given in a counterbalanced order; that is, the sequence of doses was randomized such that any one dose had an equal probability of being given first, second, third, or fourth. Rats in both the control and CPF groups and for each sex received the challenge drug doses in all the different possible sequences, and at least two days elapsed between testing with each subsequent dose.

### Data analysis.

Data are presented as means and SEs. To establish the effects of CPF and its relationship to other variables, a multivariate analysis of variance (ANOVA; data log-transformed whenever the variance was heterogeneous) was first conducted, encompassing neonatal treatment, sex, and, where multiple evaluations were run on the same animal (radial-arm maze, ketanserin challenge), the repeated measure associated with that test. Where CPF treatment interacted with the other variables, data were then subdivided to permit lower-order ANOVAs, followed, where appropriate, by Fisher’s protected least significant difference to identify individual values for which the CPF groups differed from the corresponding control. For all tests, significance for main treatment effects was assumed at *p* < 0.05; however, for interactions at *p* < 0.1, we also examined whether lower-order main effects were detectable after subdivision of the interactive variables (Snedecor and Cochran 1967).

## Results

### Elevated plus maze.

**Figure 1:**
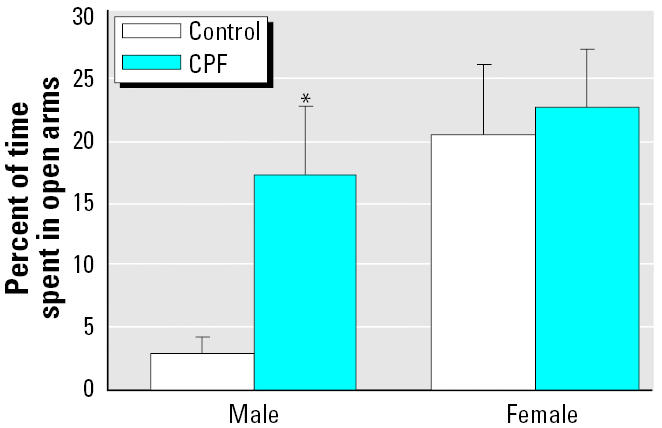
Effects of neonatal CPF exposure on performance in the elevated plus maze, presented as the percentage of time spent in the open arms. Data represent means and SEs. ANOVA indicated a main effect of CPF treatment (*p* < 0.0005) and an interaction of treatment × sex (*p* < 0.06). Separate analyses were conducted for males and females, showing a significant treatment effect only in males (asterisk). Control males and females also differ from each other significantly (*p* < 0.008).

In agreement with an earlier report (Elliott et al. 2004), control males spent a much shorter period in the open arms of the plus maze than did females (main effect of sex, *p* < 0.008; Figure 1). CPF exposure had significant, sex-selective effects, raising the open-arm time in males to the same level as found in females, whereas the CPF-treated females showed no net difference from their corresponding controls. A similar pattern was seen for locomotor activity in the plus maze, as assessed by the number of center crossings: control males (4.6 ± 0.7 crossings) were much less active than were control females (8.7 ± 1.5, *p* < 0.03 vs. males), and CPF exposure increased the male value (8.2 ± 1.4, *p* < 0.04 vs. control males) to match that of females, whereas females were unaffected by CPF treatment (9.0 ± 1.0).

### Chocolate milk preference.

**Figure 2:**
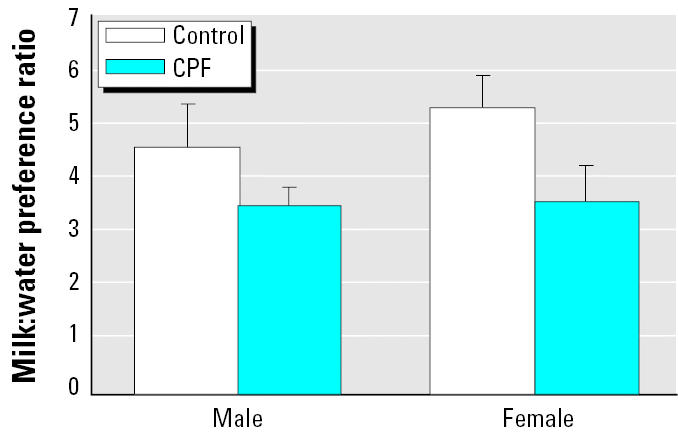
Effects of neonatal CPF exposure on the consumption of chocolate milk compared with water. Data represent means and SEs of the ratio of chocolate milk consumption to water during a 2-hr period. ANOVA indicates a main effect of CPF (*p* < 0.02) without an interaction of treatment × sex, so lower-order evaluations were not performed for males and females.

Control males and females showed equivalent preference for chocolate milk versus water in the anhedonia test. CPF treatment produced a parallel decrease in the preference ratio across both sexes (main effect of CPF, *p* < 0.02; Figure 2). There were no changes in total fluid consumption during the 2-hr test period (23 ± 1 mL for each group).

### Radial-arm maze training.

**Figure 3:**
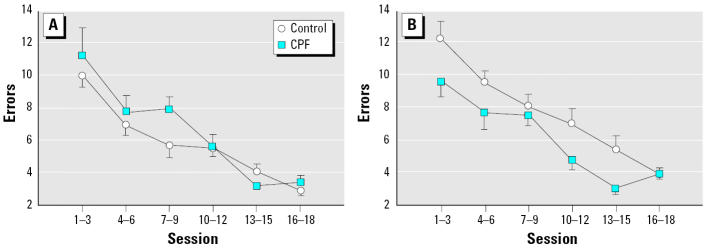
Effects of neonatal CPF exposure on working memory errors in the 16-arm radial maze in (*A*) males and (*B*) females. Values shown are means and SEs across groupings of three sessions. ANOVA indicated a significant treatment × sex interaction (*p* < 0.01), with the females showing a main treatment effect of CPF (*p* < 0.02).

As identified in previous studies (Icenogle et al. 2004; Levin et al. 2001, 2002), there was a significant sex difference in the rate of working memory errors during training in the radial-arm maze, with control males showing consistently fewer errors than did control females (*p* < 0.008; Figure 3). The effects of CPF exposure similarly showed strong sex dependence (*p* < 0.01 for the interaction of treatment × sex), reflecting a tendency to increase the error rate in males but to decrease the rate significantly in females. Accordingly, CPF exposure effectively eliminated the normal, characteristic sex difference in maze performance (no significant effect of sex in the CPF group). These findings for the training stage thus replicate previous findings in a separate cohort of animals at somewhat younger ages (Levin et al. 2001).

**Figure 4:**
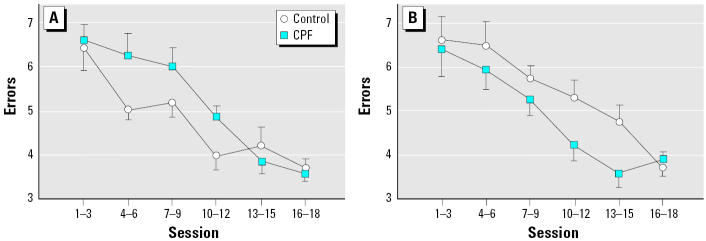
Effects of neonatal CPF exposure on reference memory errors (means and SEs) in the 16-arm radial maze in (*A*) males and (*B*) females. Values shown are means and SEs across groupings of three sessions. ANOVA indicated a significant treatment × sex interaction (*p* < 0.004). Both groups showed treatment effects, with males displaying an increase in errors (*p* < 0.03) and females a decrease (*p* < 0.06).

A similar pattern was seen for reference memory errors, with a sex difference in the control group (errors in males < females, *p* < 0.008) but not in the animals exposed to CPF. CPF treatment again evoked a significant sex-related alteration of performance (treatment × sex, *p* < 0.004). The net effect of CPF was to increase the error rate significantly in males (Figure 4A) but to cause a decrease in females that was at the margin of significance (Figure 4B). ANOVA across both working and reference memory errors confirmed that the net increase in error rate in males and decrease in females were both significant (*p* < 0.05 and *p* < 0.007, respectively).

In contrast to the effects of CPF on working and reference memory errors, there were no alterations in latency (time per arm entry; data not shown).

### Ketanserin challenges in the radial arm maze.

**Figure 5:**
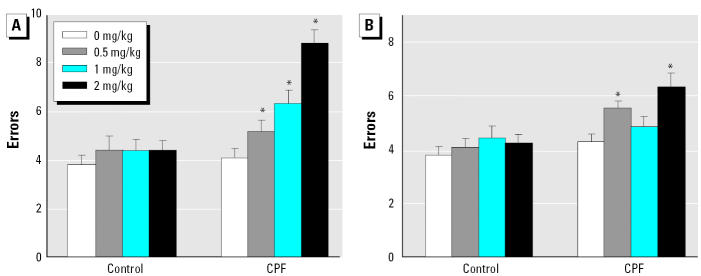
Effects of the ketanserin challenge in the 16-arm radial maze showing effects on error rates for working memory (*A*) and reference memory (*B*) at three different ketanserin doses (0.5, 1, and 2 mg/kg). Data represent means and SEs, with values for males and females combined because of the absence of an interaction of treatment × sex. In (*A*), ANOVA indicated a main effect of CPF treatment (*p* < 0.0001) and an interaction of CPF × ketanserin dose (*p* < 0.0001). In (*B*), both terms were also significant (*p* < 0.0001 and *p* < 0.002). Because of the interaction of the two treatments, separate analyses were then conducted for the effects of ketanserin in the control and CPF-treated groups. For both measures, ketanserin had no significant effect in the controls, whereas it elicited significant increases in errors in the CPF group (*p* < 0.0001 for *A*; *p* < 0.002 for *B*).
*Ketanserin doses that elicited significant increases in errors.

Despite the differences during acquisition, CPF-exposed animals achieved error rates similar to those of controls by the end of the 18 training sessions. At that point, animals were challenged with ketanserin to evaluate the contributions of 5HT_2_ receptor activity to memory. At the doses used, ketanserin challenge did not cause any amnestic effect in control rats: neither working (Figure 5A) nor reference (Figure 5B) memory error rates were affected. In contrast, in the CPF group, ketanserin evoked a significant increase in both memory parameters, with a relatively larger effect on working memory (Figure 5A) than on reference memory (Figure 5B). Unlike the effects on acquisition, the response to ketanserin challenge was not sex selective (no interaction of ketanserin × sex).

## Discussion

Animal models of depression that encompass deficiencies in 5HT synaptic function display a uniform set of behavioral parallels: locomotor hyperactivity and increased open-arm time in the elevated plus maze, anhedonia in the chocolate milk consumption test, and cognitive impairment; all of these resolve with therapies that restore 5HT function to normal (Cairncross 1984; Cryan et al. 1999; Harkin et al. 1999; Jesberger and Richardson 1985; Juckel et al. 2003; Kelly et al. 1997; Kusserow et al. 2004; Leonard and Tuite 1981; Mar et al. 2000; Martin et al. 1998; McGrath and Norman 1999; Pucilowski et al. 1993; Richardson 1991; Slotkin et al. 1999). In the present study, essentially the same pattern of behavioral defects was obtained with CPF exposure in the early neonatal period, a treatment similarly shown to produce long-term alterations in 5HT synaptic proteins commensurate with a reduction in 5HT function (Aldridge et al. 2004). Indeed, it is possible to draw a direct connection between at least one feature of the neurochemical alterations and the corresponding behavioral effect. CPF exposure evokes prominent up-regulation of 5HT_1A_ receptors (Aldridge et al. 2004), and in a recent study, transgenic animals overexpressing this receptor similarly showed increased open-arm activity in the plus maze, just as found here for the CPF group (Kusserow et al. 2004). Furthermore, the CPF effects on receptor expression (Aldridge et al. 2004) and behavior in the plus maze show the same sex selectivity (effects in males > females). Indeed, because of the role of 5HT in anxiety (Kusserow et al. 2004), the increase in plus-maze open-arm activity seen in the CPF-exposed males, an anxiolytic effect, may be directly attributable to the loss of 5HT synaptic function.

The sex differences in 5HT synaptic proteins (Aldridge et al. 2004) and in the effects seen here in the plus maze point to the potential mechanisms underlying the ability of CPF to disrupt development and function of 5HT systems. Both neurochemical and behavioral findings indicate that, when CPF exposure occurs during a developmental stage before sexual differentiation of the brain, there are no sex differences in the ultimate effects (Aldridge et al. 2004; Icenogle et al. 2004; Meyer et al. 2004b; Qiao et al. 2004), whereas similar treatments targeted to the peak period of sexual differentiation in late gestation through the early neonatal period (McCarthy 1994), produce disparate effects in males versus females (Aldridge et al. 2004; Dam et al. 2000; Garcia et al. 2003; Levin et al. 2001, 2002; Meyer et al. 2004a, 2004b; Qiao et al. 2003). CPF lacks sufficient estrogenic activity to account directly for these effects (Andersen et al. 2002), and although it interferes with testosterone catabolism (Usmani et al. 2003) and can elicit secondary endocrine alterations (Guven et al. 1999), the doses required for such actions lie above the threshold for systemic toxicity, unlike the effects on neurodevelopment. Instead, given the ability of CPF to disrupt neural cell replication, differentiation, axonogenesis, and synaptogenesis (Barone et al. 2000; Casida and Quistad 2004; Gupta 2004; Pope 1999; Slotkin 1999, 2004), it is most likely that the adverse effects of CPF on brain cell development themselves contribute to changes in sexual differentiation and resultant sex-related outcomes. In fact, our results substantiate this interpretation: in every case where the effects of CPF showed sex selectivity, the net actions represented the blunting of normal sex differences in the corresponding behavior. Thus, in the elevated plus maze, females normally show greater locomotor activity and time spent in the open arms (Elliott et al. 2004), and CPF obliterated this difference by feminizing the behavior of males, increasing their scores to those seen in normal females. In keeping with effects targeting sexual differentiation, the activation of locomotor activity in males has its onset only after puberty (Dam et al. 2000). In the same vein, in the 16-arm radial maze, males ordinarily make fewer working and reference memory errors than do females (Icenogle et al. 2004; Levin et al. 2001, 2002), and neonatal CPF exposure raised the error rate in males and decreased that of females so that there were no longer any sex differences. Our results point out the importance of distinguishing both neurochemical and behavioral outcomes in males and females, as opposed to the not atypical practice of examining only males, or of combining results from both sexes without discrimination.

It is important to note that the effects of CPF are sex selective but not sex specific; that is, whereas some of the 5HT neurochemical (Aldridge et al. 2004) and behavioral (Dam et al. 2000; Levin et al. 2001) effects are greater in males than in females, both sexes do show significant alterations. In the present study, anhedonia was detected in both sexes, and the cognitive effects of ketanserin challenge were similar in males and females. In fact, the latter test raises an interesting question: If CPF elicits deficits in 5HT synaptic function, why does 5HT_2_ receptor blockade increase error rates in the CPF-exposed animals when it has no such effect in controls? Here, it is important to note that, ordinarily, hippocampal cholinergic systems play a critical role in radial-arm maze performance, and it is these inputs that are especially targeted by neonatal CPF exposure (Slotkin et al. 2001), drastically reducing their contribution to working and reference memory performance (Levin et al. 2001). CPF-exposed animals do learn the radial-arm maze but by alternate, noncholinergic mechanisms (Levin et al. 2001), and the present results indicate that 5HT provides one of these critical adaptations. Accordingly, although 5HT function is deficient, yet at the same time, the CPF-exposed animals have an atypical reliance on 5HT inputs for behaviors that do not ordinarily depend on this transmitter. In turn, this may create a situation in which cognitive function becomes increasingly vulnerable to situations that compromise 5HT synaptic function, such as genetic, neurochemical, or external factors that foster affective disorders. Indeed, many of the new generation of antidepressant and antipsychotic medications possess 5HT_2_ antagonist properties (Anttila and Leinonen 2001; Poyurovsky et al. 2003; Tyson et al. 2004), and our results suggest that early CPF exposure could lead to a subpopulation that is vulnerable to cognitive impairment by these agents, compromising their utility. This would be particularly important if, as indicated by the present results, developmental CPF exposure affects 5HT systems in a manner commensurate with augmented risk of depression, thus increasing the potential use of antidepressant medications.

Our results indicate that the long-term alterations in 5HT synaptic neurochemistry elicited by otherwise subtoxic neonatal CPF exposure (Aldridge et al. 2004) are associated with a pattern of behavioral effects commensurate with deficient 5HT function, resembling that seen in animal models of depression (Cairncross 1984; Cryan et al. 1999; Harkin et al. 1999; Jesberger and Richardson 1985; Juckel et al. 2003; Kelly et al. 1997; Kusserow et al. 2004; Leonard and Tuite 1981; Mar et al. 2000; Martin et al. 1998; McGrath and Norman 1999; Pucilowski et al. 1993; Richardson 1991; Slotkin et al. 1999). Many of the 5HT-related effects of CPF reflect disruption of neural development during sexual differentiation of the brain, thus contributing to disparate effects in males and females. Nevertheless, deficiencies in cholinergic input to cognitive performance result in an increased reliance on 5HT mechanisms that are not ordinarily called into play in normal animals, increasing the vulnerability to 5HT blockers or other potential factors that influence 5HT activity. It has been suggested that environmental toxicants that trigger long-term alterations in the programming of 5HT function contribute to appetitive and affective disorders, and resulting increases in the incidence of obesity, diabetes, and depression (Slikker and Schwetz 2003; Toschke et al. 2002; von Kries et al. 2002). Our results with neonatal CPF exposure provide some of the first support for that hypothesis.
